# Use of Under-Vine Living Mulches to Control Noxious Weeds in Irrigated Mediterranean Vineyards

**DOI:** 10.3390/plants11151921

**Published:** 2022-07-25

**Authors:** Jose G. Guerra, Félix Cabello, César Fernández-Quintanilla, José Manuel Peña, José Dorado

**Affiliations:** 1 Instituto de Ciencias Agrarias (CSIC), Serrano, 115 B, 28006 Madrid, Spain; cesar@ica.csic.es (C.F.-Q.); jmpena@ica.csic.es (J.M.P.); jose.dorado@csic.es (J.D.); 2 Escuela Técnica Superior de Ingeniería Agronómica, Alimentaria y de Biosistemas (ETSIAAB), Universidad Politécnica de Madrid, 28040 Madrid, Spain; 3 Instituto Madrileño de Investigación y Desarrollo Rural, Agrario y Alimentario (IMIDRA), Finca El Encín, Apdo 127, 28800 Alcalá de Henares, Spain; felix.cabello@madrid.org

**Keywords:** grapevine, cover crops, *Festuca ovina*, *Pilosella officinarum*, *Plantago coronopus*, *Plantago lanceolata*, weed management, weed suppression, weed biomass

## Abstract

This article assesses the use of under-vine living mulches in Mediterranean vineyards characterized by limited water resources, one of the reasons why this agronomic practice is currently unusual in these environments. The aim of the study was to test whether the use of this alternative method in Mediterranean vineyards could suppress noxious weeds without hindering optimal vineyard development. For this purpose, four native species were selected as living mulches: *Festuca ovina*, *Pilosella officinarum*, *Plantago coronopus*, and *Plantago lanceolata*. The variables measured during three years in two different experimental farms were: (a) living mulch cover, as a possible predictor of weed suppression success; (b) weed density and weed biomass, with special attention to noxious weed species; and (c) pruning weights, measured in the last year to analyze the cumulative effect of the treatments on the grapevine vegetative growth. Our results revealed that living mulches with high cover rates (average over 70%) also showed weed suppression of up to 95%, significantly controlling the occurrence of noxious weeds such as *Erigeron canadensis*. No significant effect of the different treatments on vine vegetative growth was found, although further studies would be necessary. Based on these findings, it can be concluded that under-vine living mulches could be an efficient and environmentally friendly method for weed control in Mediterranean vineyards where irrigation is available.

## 1. Introduction

Vineyards are one of the essential crops of the Mediterranean basin, where they have occupied a central role in the landscape, culture, and economy since ancient times [1]. Indeed, Spain (961 kha), France (797 kha), and Italy (719 kha) account for 34% of the world’s vineyard area [2]. Although traditionally, vineyards have been cultivated under rainfed conditions, in recent decades, the use of irrigation has become widespread in Mediterranean vineyards [3]. In the context of climate change, given the predictions of reduced rainfall in the Mediterranean basin [4], the area occupied by irrigated vineyards is expected to increase. Although this could prevent the detrimental effects of water shortage on vineyard yields [5], it could also have other undesirable effects, such as an increase in the presence of competitive and noxious weed species [6,7]. Winegrowers have tried to eradicate weeds in vineyards through conventional management based on the recurrent application of mechanical (tillage) and/or chemical (herbicides) control methods. However, numerous studies have highlighted in recent years critical problems associated with these weed management practices, such as the emergence of herbicide-resistant species [8], soil erosion [9], or biodiversity decline [7]. This has led to a search for alternative weed management in line with the European Green Deal (EU Farm to Fork Strategy), being the use of plant covers (e.g., cover crops, spontaneous plant covers) as one of the main alternatives to conventional management. Plant covers can provide multiple ecosystem services, such as erosion reduction, increased populations of natural enemies of crop pests, or biodiversity enhancement [7,10,11,12,13], but can also have a depressive effect on grapevine vigor and vineyard yield [7,14,15], therefore, some precautions would be necessary when used in Mediterranean vineyards. Nonetheless, winegrowers are looking for a balance in vegetative growth since excessive vine growth can have negative effects in terms of reduced fruit and wine quality [16,17].

In the case of cover crops (i.e., sown plant covers), one of the main benefits widely reported is weed control [18,19,20,21], which can be strongly correlated with cover crop biomass and cover crop type. Thus, cover crop biomass is inversely related to weed biomass and weed density [22], with grasses in general providing greater weed suppression than broadleaf species [21,22]. A particular case within cover crops are living mulches, which are maintained alive throughout the crop growing season [23], usually consisting of perennial plants that form a permanent cover without requiring annual seeding [24]. The usual purpose of living mulch application has typically been weed control [25,26,27,28], being more frequent in woody crop rows, where they could provide effective weed control but not significantly affect crop yields [24]. Living mulches have been tested for years on arable and woody crops, with positive effects on soil quality [29,30,31], beneficial insects [32,33], or soil organic carbon [34,35]. Some experiments with under-vine living mulches have been documented in humid-climate vineyards, revealing, beyond the benefits mentioned above, their usefulness in controlling grapevine vigor and achieving a more balanced vegetative growth [36,37,38,39]. However, in Mediterranean climates where vineyard-producing areas are generally associated with a scarcity of water resources, their use is still very unusual [40] and plant covers, if any, are commonly limited to the vineyard inter-rows. Nonetheless, combining under-vine living mulches with irrigation application could be a suitable strategy, as plant covers, besides improving soil conditions and water infiltration, could also limit the presence of noxious weed species [7,41]; while irrigation application could reduce the negative impact of water competition due to plant covers on the vineyard [42]. Therefore, such competition between cover crops and grapevines must be carefully managed to avoid undesirable results in grape production and quality [43].

With the main objective of assessing the influence of permanent plant covers along vineyard rows on noxious weed populations as well as on vineyard growth development, a study was carried out from 2018 to 2021 in two irrigated Mediterranean vineyards using four herbaceous species as under-vine living mulches (*Festuca ovina*, *Pilosella officinarum*, *Plantago coronopus*, and *Plantago lanceolata*). We hypothesized that a permanent plant cover without specific management needs could limit the presence of noxious weeds, while not limiting the development of vines. To this end, after the experiments were implemented in 2018, in the following years, we analyzed the effect of the four living mulches on weed density and weed biomass, particularly on the suppression of noxious weed species. In addition, the effect of the different living mulches on grapevine vegetative growth was also measured. The results provided here could help to extend this alternative weed management method to other Mediterranean vineyards.

## 2. Materials and Methods

### 2.1. Study Site

The study was conducted in parallel in two experimental vineyards located in a wine-growing region in the center of the Iberian Peninsula: (A) ICA-CSIC farm “La Poveda” (Arganda del Rey, Madrid, Spain; altitude 613 m.a.s.l.); and (B) IMIDRA farm “El Socorro” (Colmenar de Oreja, Madrid, Spain; altitude 755 m.a.s.l.). The climate in the region is semi-arid continental Mediterranean, with a mean annual temperature in “La Poveda” of 15.1 °C and average annual rainfall of 369 mm, while 13.7 °C and 421 mm in “El Socorro” (data from local neighboring weather stations for the period 2001–2021). Detailed climate data for the study period are shown in Table 1. The soil type is Xerofluvent (pH 8.1) with a sandy loam texture in “La Poveda” and Calcic Haploxeralf (pH 8.4) with a clay loam texture in “El Socorro”. Grapevines (*Vitis vinifera* L. cv. Tempranillo) were grown in both fields in the Cordon Royat formation, with a planting frame of 2.5 m (inter-row spacing) × 1.2 m (inter-vine spacing) in “La Poveda”, while 2 m × 1.1 m in “El Socorro”. The vineyard inter-rows were tilled with cultivator in “La Poveda”, while mowed with a flail mower in “El Socorro”. On both farms, deficit drip irrigation was applied in the vineyard rows from May to September.

### 2.2. Plant Species Selected for Living Mulches

The key premise was to seek species that could permanently cover the soil without the need for reseeding. Thus, after an initial screening (apart from the four species chosen for this study, *Astragalus hamosus* L., *Lotus corniculatus* L., *Medicago polymorpha* L., and *Sulla coronaria* (L.) B.H.Choi & H.Ohashi were assessed), four indigenous species that are common in the Mediterranean flora were selected: *Festuca ovina* L., *Pilosella officinarum* Vaill., *Plantago coronopus* L., and *Plantago lanceolata* L. These species were chosen on the basis of their different functional traits to assess their ability to compete with other species and form monospecific covers (Table 2). *Festuca ovina* is a tuft-forming perennial grass previously used as under-vine plant cover [36], which forms a dense grass cover that could intercept weed seed rain, as described by Doisy et al. [44]. *Pilosella officinarum* is a prostrate growing plant with sexual, apomictic, and vegetative (by stolons) reproduction [45,46], which has a high colonizing potential as it is able to rapidly form monospecific patches and exclude other species [47], being considered a potentially invasive species in grasslands outside its natural range [48,49]. *Plantago coronopus* is a biennial (rarely perennial) rosette plant, while *Plantago lanceolata* is a perennial rosette species that has already been used in cover crop mixtures in vineyards [50]. Both *Plantago* species could control weed emergence by competing effectively for light and nutrients [20,23], with this competition expected to be greater in *Plantago lanceolata*, given its higher biomass. Nonetheless, their competitive ability may be conditioned by their different life cycles, i.e., perennials vs. biennials.

### 2.3. Experimental Design

The experimental design consisted of randomized blocks with four replications and six treatments: (i) an untreated control (UC); (ii) a weed-free control by manual weeding (WC) to estimate the vineyard growth development in the absence of weeds; and four under-vine living mulches, (iii) *Festuca ovina* (FO), (iv) *Pilosella officinarum* (PO), (v) *Plantago coronopus* (PC), and (vi) *Plantago lanceolata* (PL). Each treatment was distributed along eight inter-vine spacing, i.e., 9.6 m in La Poveda (8 × 1.2 m) and 8.8 m in El Socorro (8 × 1.1 m). The four selected species were planted in autumn 2018, in a strip 0.5 m wide under the vineyard row. With the exception of *Pilosella officinarum*, which was transplanted (density of 12 plants regularly distributed in the inter-vine spacing) from vegetative material collected from a nearby population, the rest of the species were sown from seed (seeding rates following the recommendations of the seed supplier: *Festuca ovina*, 9.5 g m^−2^; *Plantago coronopus*, 1 g m^−2^; *Plantago lanceolata*, 2 g m^−2^).

### 2.4. Field Data Collection

After the year of establishment, in the following three years (i.e., from 2019 to 2021) plant surveys were conducted at two different times, coinciding with the early and late crop growing season (May and September, respectively), by placing three sampling frames (0.33 × 0.33 m) in each of the 40 plots (2 fields × 4 replications × 5 treatments, excluding the weed-free control). At each sampling frame, the percentage cover of target species used as living mulch was estimated [52].

Previous studies in non-permanent cover crops have commonly used cover crop biomass as a predictor of weed suppression effect [22,53,54]. In our case, being a permanent cover crop, a non-destructive method was chosen in order not to affect plant viability, so the percentage cover of living mulches was measured as a possible predictor of weed suppression. Additionally, a rough estimation of living mulches biomass was made, as explained in Appendix A, in order to compare our results with previous studies.

Weed species (in a broad sense, all spontaneous flora) were identified from Castroviejo [55], updating the nomenclature according to International Plant Names Index [56], and weed density (number of individuals per m^2^) was recorded. All weeds at each sampling frame were collected in paper bags, dried in the laboratory (oven at 105 °C for 24 h) and weighed on a precision balance to obtain the weed biomass data.

Finally, the cumulative effect of the three years of treatments on grapevine vegetative growth was estimated. For this purpose, the pruning weights of the six central vines in each plot (i.e., excluding border vines) in each of the 48 plots (2 fields × 4 replications × 6 treatments, including the weed-free control) were measured in the last year (i.e., 2021).

### 2.5. Statistical Analysis

The effect of the treatments on the response variables was measured separately for each experimental farm using independent models because of contrasting differences (i.e., soil type, weather, etc.) between the two farms. On the one hand, the degree of establishment of different species used as under-vine living mulches was analyzed, using the vegetation cover as the response variable, treatment (FO, PO, PC, PL), and year as the fixed factors. Year was used as a fixed effect since the temporal evolution of living mulches could follow a non-random pattern specific to each species. Living mulches vegetation cover was expressed as a decimal (i.e., range 0 to 1) to fit a generalized linear model with beta distribution [57].

On the other hand, weed data were pooled from the three sampling frames per plot, thereby obtaining a matrix that included data per plot for two sampling times and for three years (40 plots × 2 sampling times × 3 years; *n* = 240). Weed density models were constructed for overall species and, individually, for those species with a significant presence. To determine which species had significant presence, the absolute weed density data were transformed into relative density:

$$\mathrm{Relative}\;\mathrm{density}=\frac{\mathrm{number}\;\mathrm{of}\;\mathrm{individuals}\;\mathrm{of}\;a\;\mathrm{species}\;\mathrm{on}\;a\;\mathrm{plot}\;x}{\mathrm{total}\;\mathrm{number}\;\mathrm{of}\;\mathrm{individuals}\;\mathrm{on}\;\mathrm{plot}\;x}\times 100$$

A significant presence was thus established in this work for those species with relative density >3%. The effect of the different treatments (UC, FO, PO, PC, PL) on weed density and weed biomass was estimated using generalized linear mixed models, fitted with a negative binomial distribution for weed density and a gamma distribution for weed biomass. In total weed density and weed biomass models, the year was used as a fixed effect, considering that both total density and biomass of species depend on living mulches cover, which in turn depends on the year factor. However, in the individual species density models, the year was considered a random effect since the higher or lower density of a species can be driven by stochastic processes. In general, block was used as a random effect. The relationship of weed density and weed biomass (response variables) with living mulches cover was explored using linear regression models and Spearman’s correlation test. In the same way, the relationship between living mulch cover and the ratio weed biomass/density was analyzed. The lower this ratio, the lower the average biomass of weeds present in a plot. This correlation could provide an analysis of whether weed biomass per plant unit increases or decreases as a function of living mulch cover.

Additionally, a linear mixed model was constructed to measure the effect of the different treatments (WC, UC, FO, PO, PC, PL) on grapevine vegetative growth, including block as a random effect. A logarithmic transformation (log[*x* + 1]) was performed to fit the response variable to a normal distribution. The relationship between grapevine vegetative growth and weed biomass was measured using a Spearman correlation test.

All statistical analyses were performed in R 4.1.0. Beta regression models were fitted with the *betareg* package in R [58], estimating its fit by pseudo *R*^2^. Generalized linear mixed models were fitted with the *GLMMtmb* package [59], computing marginal *R*^2^ (fixed effects variance) and conditional *R*^2^ (full model variance) using the *performance* package [60]. The corrected Akaike Information Criterion (AICc) was applied to screen the best models, selecting those with the lowest AICc score. For all models, the statistical significance of the fixed effects was estimated by a type III ANOVA test. To compare the mean of the levels of one factor, the Student’s *t*-test was performed, adjusting the *p*-values with the Bonferroni correction.

## 3. Results

### 3.1. Establishment of Under-Vine Living Mulches

**Figure 1:**
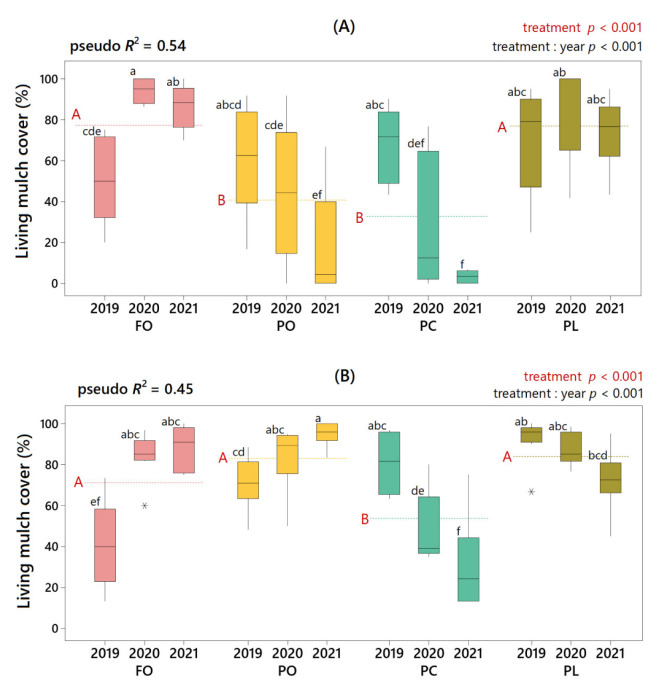
Living mulch cover (%) according to treatment and year on farm A (**A**) and farm B (**B**). Dashed lines mark the three-year mean for each treatment (FO, *Festuca ovina* living mulch; PO, *Pilosella officinarum* living mulch; PC, *Plantago coronopus* living mulch; PL, *Plantago lanceolata* living mulch). Significance levels (*p*-values) of the factor “treatment” and of the interaction between “treatment” and “year” are indicated in the text in red and black ink, respectively. For the “treatment” level, means were significantly different if red capital letters next to the dashed lines are different. For the “treatment: year” level, boxes with different lower-case black letters had significantly different means. In the upper left corner, pseudo *R*^2^ indicates the variance explained by the beta regression model.

The percentage cover observed in living mulches varied according to the species used, the year and the farm studied (Figure 1). Living mulches of *Festuca ovina* and *Plantago lanceolata* (hereinafter, FO living mulch and PL living mulch) were successfully established on the two farms studied, showing an average cover (over the three years) of about 80% on both farms. In the case of FO living mulch, although in the first year the percentage of cover barely exceeded 40%, from the second year onwards, it reached cover close to 100%. Regarding PL living mulch, no significant inter-annual variations were found, although a decrease in the percentage cover was observed in the third year. The living mulch of *Plantago coronopus* (hereinafter, PC living mulch) was successfully established in the first year but showed a drastic decrease in the percentage cover from the second year onwards, thus registering in the whole three years and the two farms with the lowest establishment values compared to the other living mulches. In the case of living mulch of *Pilosella officinarum* (hereinafter, PO living mulch), the observed response was clearly different between farms. On farm “La Poveda” (hereinafter, farm A), although in the first year the percentage cover did not differ significantly from that observed for the other species, from the second year onwards, a decline began, which was significantly marked in the third year, when in some plots the percentage cover dropped below 10%. In contrast, the percentage cover of PO living mulch on farm “El Socorro” (hereinafter, farm B) increased over the years, from 68% to 95% average cover from 2019 to 2021, respectively.

### 3.2. Effect of Under-Vine Living Mulches on Weed Density

A total of 59 plant species were identified during the three years of weed sampling, 35 on farm A and 42 on farm B, of which 18 species were present on both farms (Table 3). On farm A, the most abundant species were *Erigeron canadensis* (relative density of 39%), *Bromus madritensis* (13%), *Cyperus rotundus* (12%), and *Lolium arundinaceum* (12%). Weed community composition was different on farm B, with *Euphorbia prostrata* (52%), *Convolvulus arvensis* (13%), *B. madritensis* (7%), and *Sonchus asper* (6%) as the most abundant species.

**Figure 2:**
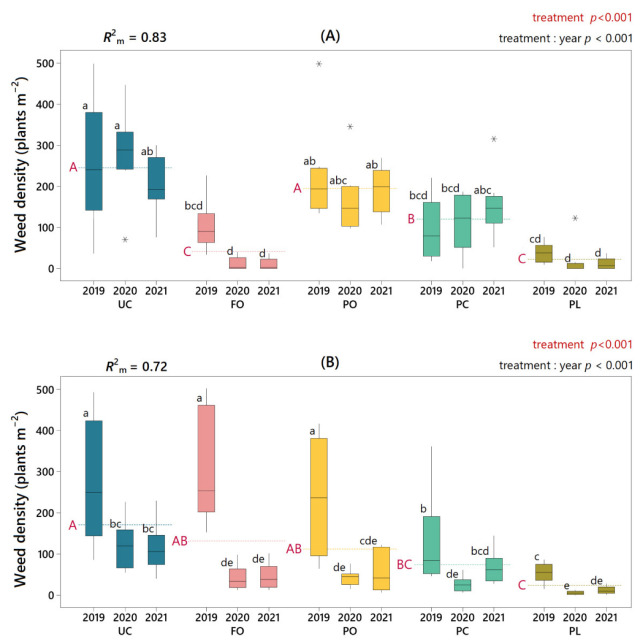
Weed density (plants m^−2^) according to treatment and year on farm A (**A**) and farm B (**B**). Dashed lines mark the three-year mean for each treatment (UC, untreated control; FO, *Festuca ovina* living mulch; PO, *Pilosella officinarum* living mulch; PC, *Plantago coronopus* living mulch; PL, *Plantago lanceolata* living mulch). Significance levels (*p*-values) of the factor “treatment” and of the interaction between “treatment” and “year” are indicated text in red and black ink, respectively. For “treatment” level, means were significantly different if red capital letters next to the dashed lines are different. For “treatment: year” level, boxes with different lower-case black letters had significantly different means. In the upper left corner, *R*^2^_m_ indicates the variance explained by the fixed effects of the mixed model. Outliers are indicated with an asterisk (*).

The use of living mulches had a significant effect on weed density, although the effect varied depending on the mulching species used and the weed species to be controlled. On farm A, FO and PL living mulches drastically reduced total weed density compared to the control, and a smaller but significant reduction in PC living mulch was observed (Figure 2). On farm B, only the two *Plantago* species showed a significant reduction in total weed density, with the suppression by PL living mulch being particularly marked.

When the year factor was considered (i.e., treatment: year), different patterns were shown according to the living mulch species (Figure 2). For example, a regular behavior in all three years, either because showing significant differences with respect to untreated control (hereinafter UC), e.g., in PL living mulch on both farms and FO living mulch on farm A, or because showing no significant differences with respect to UC, e.g., in PO living mulch on farm A. However, other remarkable patterns were found in species such as PC living mulch, which showed a significant reduction in weed density only during the first two years, then losing this weed-suppressive capacity in the third year on both farms. An opposite pattern was observed in FO living mulch on farm B, since during the first year had no effect on weeds (UC = 279 plants m^−2^; FO = 308 plants m^−2^), but from the second year onwards, drastically reduced the total weed density.

The effect of living mulches on the density of weed species with a significant presence (i.e., relative density > 3%) is shown in Table 4. On farm A, the four living mulches significantly reduced the presence of the noxious weed *E. canadensis* (ERICA), especially FO and PL living mulches, with weed suppression of 92% and 97%, respectively. The density of *S. asper* (SONAS) was significantly reduced by PL living mulch on both farms, by FO living mulch on farm A, and by PO and PC living mulches on farm B. Noxious weed *C. arvensis* (CONAR) was also significantly suppressed by PL living mulch on farm B.

Beyond the effect on noxious weeds, FO and PL living mulches significantly reduced the density of *B. madritensis* (BROMA) on both farms. In addition, PL living mulch significantly reduced the occurrence of the most abundant species on farm B, *Euphorbia prostrata* (EPHCH).

### 3.3. Effect of Under-Vine Living Mulches on Weed Biomass

**Figure 3:**
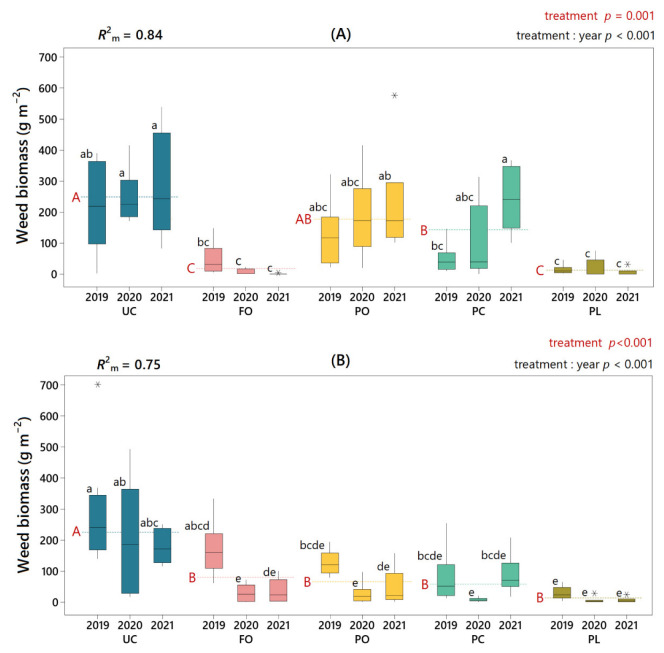
Weed biomass (g m^−2^) according to treatment and year on farm A (**A**) and farm B (**B**). Dashed lines mark the three-year mean for each treatment (UC, untreated control; FO, *Festuca ovina* living mulch; PO, *Pilosella officinarum* living mulch; PC, *Plantago coronopus* living mulch; PL, *Plantago lanceolata* living mulch). Significance levels (*p*-values) of the factor “treatment” and of the interaction between “treatment” and “year” are indicated in text in red and black ink, respectively. For the “treatment” level, means were significantly different if red capital letters next to the dashed lines are different. For the “treatment: year” level, boxes with different lower-case black letters had significantly different means. In the upper left corner, *R*^2^_m_ indicates the variance explained by the fixed effects of the mixed model. Outliers are indicated with an asterisk (*).

Similar to that observed for weed density, the results of this study have shown how the use of different living mulches largely explains the observed variance in weed biomass in both farm A (*R*^2^_m_ = 0.84) and farm B (*R*^2^_m_ = 0.75) (Figure 3). Considering the three-year average, FO and *Plantago* (i.e., both PC and PL) living mulches significantly reduced weed biomass on farm A compared to the control, with an average reduction of 95% for PL living mulch (UC = 248.64 g m^−2^; PL = 12.25 g m^−2^) and 93% for FO (UC = 248.64 g m^−2^; FO = 18.07 g m^−2^). On farm B, a significant reduction was observed for all four mulching species, particularly in PL living mulch, which showed a 93% reduction in weed biomass compared to the control (UC = 204.59 g m^−2^; PL = 13.73 g m^−2^).

The analysis accounting for the year factor (i.e., treatment: year) showed that weed biomass reduction by FO living mulch on both farms was not statistically significant in the first year but was significant in the following two years. Regarding PC living mulch, weed biomass reduction was only significant in the first two years on farm B. Regular patterns throughout the years were found for the other two species used as living mulches, with significant differences with respect to UC in PL on both farms and PO on farm B, while no significant differences in PO on farm A.

### 3.4. Relationships between Living Mulch Cover, Weed Density and Weed Biomass

**Figure 4:**
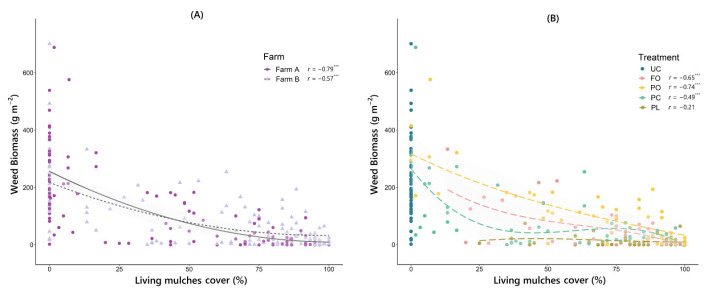
Correlations between living mulch cover (%) and weed biomass (g m^−2^) considering the factor “farm” (**A**) and the factor “treatment” (**B**). Spearman’s correlation coefficient (*r*) and significance level (no asterisks, non-significant; *** *p* < 0.001) are indicated for each of the estimated correlations. UC, untreated control; FO, *Festuca ovina* living mulch; PO, *Pilosella officinarum* living mulch; PC, *Plantago coronopus* living mulch; PL, *Plantago lanceolata* living mulch.

Overall, negative correlations were observed between living mulch cover and weed density and weed biomass, being clearly stronger on farm A than farm B and more robust when weed biomass was considered as a response variable (Table 5). These relationships were consistent as a similar response was observed on both farms. Indeed, a strong negative correlation was observed between living mulch cover and weed biomass in both farm A (*r* = −0.79) and farm B (*r* = −0.57), which exhibited a better fit using a quadratic regression models, where the slope of the curve flattens out at cover percentages above 75% (Figure 4A). However, a different response depending on the type of living mulch was found when studying the relationship between weed biomass and living mulch cover (Figure 4B).

**Figure 5:**
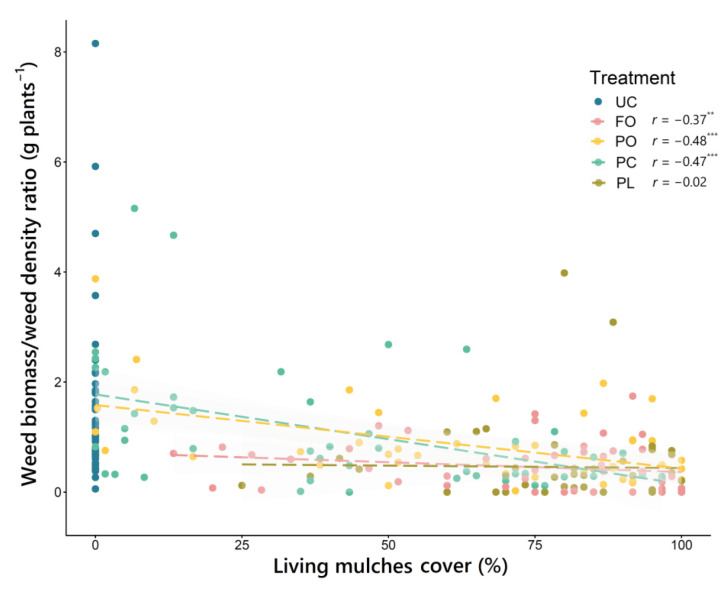
Correlation between living mulch cover (%) and weed biomass/weed density ratio (g plants^−1^) according to the factor “treatment”. Spearman’s correlation coefficient (*r*) and significance level (no asterisks, non-significant; ** *p* < 0.01; *** *p* < 0.001) are indicated for each of the estimated correlations. UC, untreated control; FO, *Festuca ovina* living mulch; PO, *Pilosella officinarum* living mulch; PC, *Plantago coronopus* living mulch; PL, *Plantago lanceolata* living mulch.

While FO and PO living mulch covers were strongly correlated with weed biomass, this relationship was weaker in the two *Plantago* species, especially in PL living mulch, where the observed correlation was not even significant. These differentiated responses therefore conditioned the average weed suppression reached by each living mulch. For example, PC living mulch resulted in high weed suppression (71%) with intermediate levels of cover (54%) on farm B, while PO living mulch required much higher cover (83%) to reach a similar level of suppression (68%) (Table 6). When the correlation between living mulch cover and the ratio weed biomass/weed density was analyzed, contrasting responses were also observed according to the type of living mulch (Figure 5). In this case, it was the FO and PL living mulches that showed a weaker correlation, exhibiting, on average, a markedly lower ratio than that observed for PO and PC living mulches (Table 6).

### 3.5. Effect of Under-Vine Living Mulches on Grapevine Vegetative Growth

**Figure 6:**
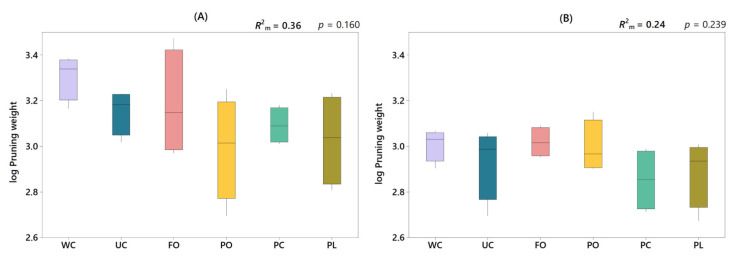
Grapevine vegetative growth (log pruning weight) according to the different treatments applied on farm A (**A**) and farm B (**B**): WC, weed-free control; UC, untreated control; FO, *Festuca ovina* living mulch; PO, *Pilosella officinarum* living mulch; PC, *Plantago coronopus* living mulch; PL, *Plantago lanceolata* living mulch. Marginal R-squared (*R*^2^_m_) indicated in the upper right corner, is the variance explained by the fixed factors of the model, in this case by “treatment”.

**Figure 7:**
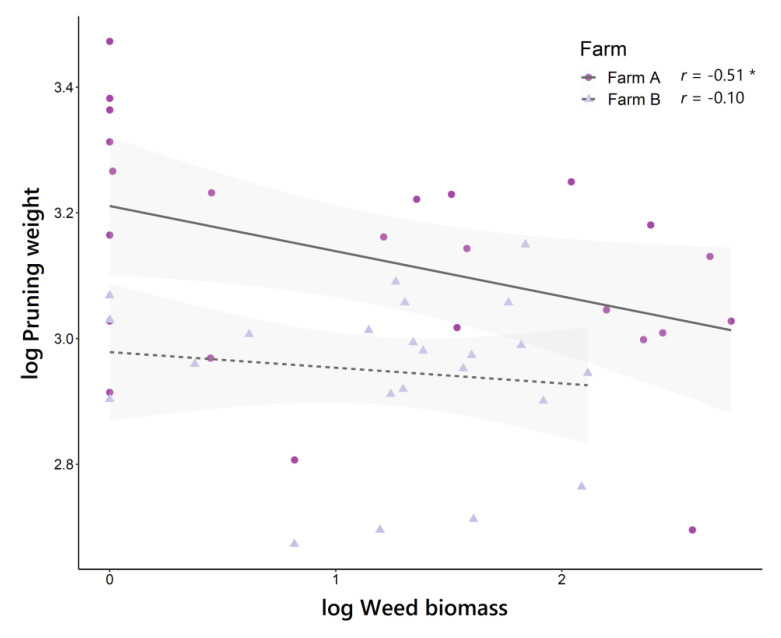
Correlation between weed biomass (log weed biomass) and grapevine vegetative growth (log Pruning weight) according to the factor “farm”. Spearman’s correlation coefficient (*r*) and significance level (no asterisks, non-significant; * *p* < 0.05) are indicated for each of the estimated correlations. Confidence intervals (95%) are shown with light-grey shaded areas.

The different treatments applied explained part of the variance observed in grapevine vegetative growth (based on pruning weights), both on farm A (*R*^2^_m_ = 0.36) and farm B (*R*^2^_m_ = 0.24) (Figure 6). However, although a slight reduction in pruning weights was observed in living mulches compared to weed-free control (WC) on both farms, there were no significant differences between treatments. An analysis of the relationship between grapevine vegetative growth and weed biomass showed a negative correlation between the two variables, with pruning weight being lower the higher the weed biomass (Figure 7). However, this correlation was only significant on farm A (*r* = −0.51*), while on farm B, it was clearly weaker (*r* = −0.10).

## 4. Discussion

### 4.1. Extend of Establishment of Under-Vine Living Mulches

Our results revealed that the living mulches with the most successful establishment were PL and FO living mulches on farm A, and PL and PO living mulches on farm B. In general, similar patterns of establishment were found in both farms for FO, PC, and PL living mulches, which suggests that they were more influenced by intrinsic traits of each species than by differential environmental factors of each farm. Indeed, FO living mulch had a limited establishment in the first year (50.9% on farm A, 41.1% on farm B), but in the second and third year they reached coverages above 80%, regardless of the farm. This is consistent with previous studies showing that *Festuca ovina* is a slow-growing species with slow establishment [61] and with a lower emergence rate than *Plantago* species used as living mulches [62]. On the other hand, the decrease in PC living mulch cover from the second year onwards probably occurred because *Plantago coronopus* is a biennial species; this study further found that spontaneous regeneration from self-seeding was very limited. Seedling recruitment of *Plantago coronopus* is strongly conditioned by rainfall [63,64], so low rainfall during the October 2020–May 2021 period (see Table 1) may have limited the recruitment of new seedlings on both farms. A lower recruitment was observed on farm A (21.4% less cover of PC living mulches than on farm B), which could be due to the lower rainfall received and a lower water retention capacity of the sandy loam soil in this farm. In contrast, the perennial species of the same genus *Plantago lanceolata*, showed a high cover on both farms during the three years of observations. On this basis, selecting perennial species as living mulch would seem more appropriate since they are able to achieve successful establishment without reliance on the success of seeding and recruitment, as these processes, in turn, depend on environmental conditions that are difficult to manage.

With regard to PO living mulches, contrasting differences were observed in the percentage of cover recorded on both farms (a 77.8% higher cover on farm B than on farm A in the last year). *Pilosella officinarum* is a drought-tolerant species that, however, does not seem to tolerate high temperatures [45]. In fact, in the Iberian Peninsula it is part of dry grasslands of cool environments, being mainly found in mountain areas [55]. The presence of a natural population of *P. officinarum* on farm B suggested that its establishment could be successful, at least on this farm, which was later confirmed in the results. Conversely, our hypothesis for farm A is that the higher temperatures reached in the summer period, more than 2 °C higher than on farm B (Table 1), could have limited their survival. Accordingly, under a global warming scenario, the use of PO living mulches in our latitudes should be limited to areas with cooler temperatures where the survival of *P. officinarum* is assured.

### 4.2. Weed Suppression by Under-Vine Living Mulches

This three-year study has shown that, as expected, successfully established living mulches (i.e., high percentage cover) generally showed high weed suppression, in accordance with previous studies on permanent living mulches [24]. The living mulch of *Plantago lanceolata* showed the highest suppression capacity, higher than in the grass living mulch *Festuca ovina* in both farms, which challenges the previous assumption that grass cover crops provide more suppression than broadleaf species [21,22]. This prior assumption may be true for broadleaf species commonly used as cover crops (i.e., brassicas, legumes) but should not be generalized. In addition, our results revealed that the extent of living mulch cover was a good predictor of weed suppression, being inversely related to weed density and biomass, regardless of the farm. This is in line with Steinmaus et al. [65], who found a similar relationship between living mulch cover and weed suppression. In light of this, our assumption is that the higher the percentage of soil covered by living mulches, the less surface area available for weeds to colonize, germinate, and establish. Nevertheless, the relationship between living mulch cover and weed biomass varied depending on the species used as living mulch (Figure 4B). Thus, the weed suppression success of PO and FO living mulches was highly dependent on the coverage achieved, but living mulches of *Plantago* were less cover-dependent, especially PL living mulch, for which no significant correlation was observed. As discussed below, these differences could be due to the different suppression mechanisms of each species. In the case of PO living mulch, the small growth habit of *Pilosella officinarum* may limit its ability to compete for light and generate a shading effect, a key feature for weed suppression [20,66,67]. Therefore, the success of this species in suppressing weeds depends to a large extent on achieving high percentages of cover, as any gap in this living mulch provides a good chance for weeds to establish and thrive. The living mulch of *Festuca ovina* was also strongly cover-dependent, showing high weed suppression at high cover levels. However, its slow establishment generating only moderate cover during the first year may compromise weed suppression in this year of implantation. Once established and due to the dense tufted cover that this species forms, we consider that it can be very effective in intercepting seed rain [44] and suppressing weeds by shading, thus limiting weed seed germination and weed seedling growth. In fact, the low ratio observed between weed biomass and density in FO living mulch (Table 6) may be indicative of this suppressive effect on seedling growth. Similarly, a very low ratio was also found in PL living mulch. Both species (i.e., *Festuca ovina* and *Plantago lanceolata*) showed similar biomass and height, clearly higher than the other two species used (see Table 2). These traits could be considered a differential factor in terms of increased competition for light (the aforementioned “shading effect”) and nutrients against weeds, in line with previous work indicating that cover crop biomass is a key driver of weed suppression [22,53,54]. However, all the above does not explain why PL living mulch, with a biomass similar to that of FO living mulch, showed a higher weed suppression capacity compared to other living mulches even at intermediate cover levels (Figure 4B). A plausible explanation could lie in a physical mechanism associated with leaf traits, whereby the leaves of *Plantago lanceolata*, with a significantly larger leaf area than the others (Table 2), could shade a greater soil surface, therefore, a medium cover would be enough to exert a competitive shading. On the other hand, the presence of phytotoxic compounds has been cited in *Plantago lanceolata* [68] and other species of the genus [69], so that weed suppression could be partly related to allelopathic effects, although this could not be demonstrated in our study. In this sense, a lower relationship between weed biomass and leaf mass cover was observed in PC living mulch, achieving higher weed suppression than FO and PO living mulches at cover levels below 70% (Figure 4B). Given its growth habit and leaf size, we consider it unlikely that this is due to an increased shading effect, but possibly by an allelopathic effect.

### 4.3. Control of Noxious Weeds by Under-Vine Living Mulches

Our results indicated that the use of under-vine living mulches can drastically limit the occurrence of *E. canadensis*, as well as other problematic weeds in vineyards, such as *Convolvulus arvensis* or *S. asper* (Table 2). *Erigeron canadensis* is a highly competitive weed that poses a serious threat, given its resistance to glyphosate [70,71] and its depressive effect on grapevine vegetative growth [72]. This species was significantly controlled by all living mulches, especially by FO and PL living mulches (92% and 97% suppression), similar to the suppression levels found by Pittman et al. [73] in cover crops mixtures. Considering that germination of *E. canadensis* is highly light-dependent [74,75], our assumption is that *Festuca ovina* and *Plantago lanceolata*, with the highest shading capacity, were also the most effective in suppressing this noxious weed. This is in line with Steinmaus et al. [65], who observed that the use of living mulches in a Californian vineyard achieved high weed suppression due to light interception. In addition, Doisy et al. [44] have reported that grass covers (*Festuca arundinacea* Schreb and *Dactylis glomerata* L.) intercept 56% of seed rain of *E. canadensis*, which could likewise occur in FO living mulch. Similar effects of grass cover were reported on *S. asper* with 66% interception of seed rain [44], a species that also has a highly dependent germination on light received [74]. Therefore, as discussed for *E. canadensis*, we consider that these mechanisms could explain to a great extent the high suppression of *S. asper* by FO living mulch on farm A and by PL living mulch on both farms. The low suppression of *S. asper* by FO living mulch on farm B could be due, as discussed above, to the low establishment of *F. ovina* during the first year. This fact might also have limited the suppressive capacity of FO living mulch on *C. arvensis*, which was only significantly controlled by PL living mulch on farm B, with a 72% reduction with respect to control. This result is particularly relevant given that *C. arvensis* is a perennial geophyte, persistent and hard to manage, which can cause significant crop losses [76]. Our results contrast with the findings of Steinmaus et al. [65], as the living mulches used by them (*Avena sativa* L. and *Vicia benghalensis* L.) not only failed to control *C. arvensis*, but increased its dominance. Nonetheless, our results do not clarify the mechanism involved in the suppression of *C. arvensis* by PL living mulch, which could be due to a combination of all the mechanisms that have been discussed above.

Likewise, it should be noted that the two most abundant species in the vineyards studied were two invasive alien species: the aforementioned *E. canadensis* and *Euphorbia prostrata*, although the latter does not a priori pose any threat to grapevines. The application of irrigation in Mediterranean woody crops during the summer months can lead to the proliferation of alien weeds [77,78]. In the current context, in which the surface area occupied by irrigated vineyards in Mediterranean countries such as Spain is increasing exponentially [79], irrigated vineyard rows could be a potential reservoir of invasive alien weeds, some of which, such as *E. canadensis* or other species of the genus *Erigeron*, could be noxious grapevine weeds. Based on our results, the use of under-vine living mulches could be a successful option to avoid the proliferation of these species.

### 4.4. Effect of Under-Vine Living Mulches on Grapevine Vegetative Growth

Our results revealed no significant effect of the different treatments on grapevine vegetative growth, which contrasts with previous studies that observed a significant effect of under-vine living mulches on vegetative growth [37,38]. However, in these studies focusing on under-vine living mulches, the treatment is the same in both vineyard rows and inter-rows, similar to what Giese et al. [36] and Vanden Heuvel and Centinari [80] correctly referred to as “complete vineyard floor cover crops”. However, in other studies in which, like us, they applied the same management in vineyard inter-rows [39,40,81], they found no significant effect of the under-vine living mulches on vine vegetative growth. Notably, when under-vine living mulches are used in newly planted vineyards, a negative effect on vegetative growth can occur [82,83], even if the same management was applied in all vineyard inter-rows. Presumably, young vines with a less developed root system seem to be more susceptible to competition from plant cover [84]. Conversely, in studies contrasting different treatments in the vineyard rows (e.g., herbicides, tillage vs. cover crops) while keeping the same management on vineyard rows, a significant effect on pruning weights was found [42,43,85].

Considering the above, our assumption is that management of vineyard inter-rows (in our case, up to five times more area than the rows) could have a significant effect on grapevine vegetative growth, proportionally greater than the effect of management on the vineyard rows. Therefore, when management in the vineyard inter-rows is the same, homogeneous results of vegetative growth in mature vineyards are to be expected, regardless of the treatment applied in the vineyard rows. In this regard, although no significant differences were observed between treatments, farm A (with tilled inter-rows) showed, on average, greater pruning weights than farm B (with mown inter-rows). This is fully consistent with the results observed in a recent study on farm B [7], in which the authors found that the percentage of bare soil (considering both vineyard rows and inter-rows) is inversely related to vineyard yield, but also to grapevine vegetative growth (unpublished data). Thus, as in the present study, management with tilled inter-rows (higher percentage of bare soil) showed significantly higher pruning weights than management with mown inter-rows (lower percentage of bare soil).

On the other hand, our results also showed a slight negative correlation between weed biomass and grapevine vegetative growth, although it was only significant on farm A. For the above reasons, we assume that the effect of management on vineyard inter-rows attenuated any possible relationships between these two variables. Moreover, considering the higher weed biomass on farm A, associated with a high presence of the noxious weed *E. canadensis*, could justify the stronger correlation observed on farm A with respect to farm B, the latter with a clearly lower impact of weeds on the vegetative growth of the vineyard. Even so, the observed correlation supports the premise that control of weed biomass under vines may play a relative role in regulating grapevine vegetative growth.

### 4.5. Implications for Vineyard Weed Management

To our knowledge, under-vine living mulches have so far been a very unusual management in Mediterranean vineyards, being limited to experimental studies [40]. However, our study suggests that permanent under-vine living mulches can be successfully established in a semi-arid Mediterranean climate, suppressing noxious weeds without jeopardizing the grapevine vegetative growth. In conditions of water scarcity, it seems daring to consider keeping the whole vineyard surface (i.e., row and inter-row) with a permanent vegetation cover. Since the competition of plant cover with the vineyard will largely depend on the percentage of bare soil [7], and this percentage can be adapted according to soil properties to avoid competition for water [86], therefore a reasonable option would be to use permanent living mulches in irrigated vineyard rows, where there could be a higher occurrence of noxious grapevine weeds [7], and keeping free of vegetation the inter-row space through soil management that prevents competition with the vineyard as much as possible.

Finally, under-vine living mulches have a low establishment cost (see Appendix B) and, as has been demonstrated here, can suppress weeds for three years without the need for mechanical or chemical operations, reducing the economic and environmental costs as well as damage to vines associated with conventional weed management. It would be advisable to study their evolution on a longer time scale, comparing unmanaged living mulches and living mulches with minimal management (e.g., annual mowing, basic fertilizer needs, etc.), which could contribute to the maintenance of the living mulch and, consequently, weed suppression over time. For a large-scale extension to commercial vineyards, it would also be necessary to develop more efficient sowing methods based on improved vineyard seeders that can adapt to both small herbaceous seeds and the reduced dimensions of the inter-vine spacing.

## Figures and Tables

**Table 1:** Mean temperature (*T*_mean_) and cumulative rainfall (R) on the two experimental farms (La Poveda, farm A; El Socorro, farm B) during the study period, grouped in two intervals: the summer season (June to September) and the rest of the year (October to May). The percentage variation of accumulated precipitation (ΔR) in each time interval with respect to the average precipitation value (data for the period 2001–2021) for the same interval is also indicated.

	Farm A			Farm B		
	*T*_mean_ (°C)	R (mm)	ΔR (%)	*T*_mean_ (°C)	R (mm)	ΔR (%)
October 2018–May 2019	11.2	172.0	−45	9.7	240.4	−33
June–September 2019	24.8	150.0	177	22.4	68.6	11
October 2019–May 2020	12.2	318.4	1	10.3	424.4	18
June–September 2020	24.3	21.0	−61	22.0	73.2	19
October 2020–May 2021	11.0	220.6	−30	9.4	286.2	−20
June–September 2021	24.2	123.6	129	21.9	149.4	142

**Table 2:** Traits of the plant species used as living mulches. Trait data have been compiled from FAWC Database [6]. Biomass estimation is described in Appendix A. RLF, Raunkiær life form; PHV, plant height (vegetative); LA, leaf area; LM, leaf mass; LDMC, leaf dry matter content, SLA, specific leaf area; CSR, plant CSR strategy according to Hodgson et al. [51].

	EPPO Code	Biomass	RLF	PHV	LA	LM	LDMC	SLA	CSR
		(g m^−2^)	-	(cm)	(mm^2^)	(g)	(mg g^−1^)	(mm^2^ mg^−1^)	-
*Festuca ovina*	FESOV	1083.2	H	17.1	46.7	1.9	422.1	14.0	S/CSR
*Pilosella officinarum*	HIEPI	256.8	H	7.3	258.8	18.9	207.0	16.0	S/CSR
*Plantago coronopus*	PLACO	491.3	H/Th	10.2	195.9	13.4	124.8	18.3	SR/CSR
*Plantago lanceoalata*	PLALA	1102.8	H	18.3	1653.3	95.4	178.6	18.9	SC/CSR

**Table 3:** List of weeds present on the two study farms during the two sampling dates, May and September (average of the three years 2019 to 2021), ranked in order of highest to lowest relative density. Those species classified as noxious weeds are indicated with an asterisk next to the EPPO code. The + and – signs indicate that the relative density of the species was less than 0.01% (+) or that the species was not found (−).

EPPO Code	Scientific Name	Farm A		Farm B	
		May	September	May	September
EPHCH	*Euphorbia prostrata* Aiton	0.01	0.02	0.25	0.71
ERICA *	*Erigeron canadensis* L.	0.43	0.35	−	−
BROMA	*Bromus madritensis* L.	0.20	0.07	0.11	0.04
CONAR *	*Convolvulus arvensis* L.	0.01	0.01	0.11	0.14
CYPRO *	*Cyperus rotundus* L.	0.05	0.17	−	−
FESAR	*Lolium arundinaceum* (Schreb.) Darbysh.	−	0.21	−	−
SONAS *	*Sonchus asper* (L.) Hill	0.06	0.01	0.11	0.03
GALPR	*Galium parisiense* L.	0.01	−	0.13	−
CYNDA *	*Cynodon dactylon* (L.) Pers.	0.05	0.06	−	−
DIPER *	*Diplotaxis erucoides* (L.) DC.	−	−	0.07	0.03
MEDMI	*Medicago minima* (L.) Bartal.	−	+	0.09	+
CHEAL *	*Chenopodium album* L.	0.03	0.03	−	−
BRODI *	*Bromus diandrus* Roth	0.04	−	0.01	−
LACSE*	*Lactuca serriola* L.	0.01	+	0.02	0.01
SOLNI *	*Solanum nigrum* L.	0.02	0.02	−	+
SETVI	*Setaria viridis* (L.) P.Beauv.	0.02	0.01	−	−
ANGAR	*Lysimachia arvensis* (L.) U.Manns & Anderb.	0.01	0.01	−	−
POLAV	*Polygonum aviculare* L.	+	+	−	0.02
LOLRI	*Lolium rigidum* Gaudin	0.02	−	0.01	−
SONOL *	*Sonchus oleraceus* L.	+	−	+	−
HORMU	*Hordeum murinum* L.	+	−	0.02	−
MEDOR	*Medicago orbicularis* (L.) Bartal.	-	−	0.02	−
ASAHA	*Astragalus hamosus* L.	+	−	0.01	−
CIRAR *	*Cirsium arvense* (L.) Scop.	−	−	+	0.01
AMAAL *	*Amaranthus albus* L.	+	+	+	+
POROL	*Portulaca oleracea* L.	−	0.01	−	−
SASKA *	*Salsola kali* L.	+	+	−	+
AVEST	*Avena sterilis* L.	−	−	0.01	−
ECHCG	*Echinochloa crus-galli* (L.) P.Beauv.	−	+	−	−
BROTE	*Bromus tectorum* L.	−	−	+	−
FILPY	*Filago pyramidata* L.	−	−	+	−
PAPRH	*Papaver rhoeas* L.	+	+	−	+
LPHCR	*Rostraria cristata* (L.) Tzvelev	+	−	+	−
MALSI *	*Malva sylvestris* L.	−	−	+	+
TRKMO	*Medicago monspeliaca* (L.) Trautv.	−	−	+	−
VEBOF	*Verbena officinalis* L.	−	−	+	−
VLPMY	*Festuca myuros* L.	−	−	+	−
HEOEU	*Heliotropium europaeum* L.	+	+	−	−
GERMO	*Geranium molle* L.	−	−	+	+
FUMOF	*Fumaria officinalis* L.	+	−	−	−
VERHE	*Veronica hederifolia* L.	+	−	−	−
CENME	*Centaurea melitensis* L.	−	−	+	−
CVPVT	*Crepis vesicaria subsp. taraxacifolia* (Thuill.) Thell.	−	−	+	−
LAMAM	*Lamium amplexicaule* L.	−	−	+	−
SCVLA	*Scorzonera laciniata* L.	−	−	+	−
TROPS	*Tragopogon porrifolius* L.	−	−	+	−
ASAST	*Astragalus stella* L.	−	−	+	−
BRORU	*Bromus rubens* L.	−	−	+	−
ERXCA	*Eryngium campestre* L.	−	−	+	−
HESCO	*Sulla coronaria* (L.) B.H.Choi & H.Ohashi	−	−	+	−
TANCR	*Taeniatherum caput-medusae* (L.) Nevski	−	−	+	−
TAROV	*Taraxacum obovatum* (Willd.) DC.	−	−	+	−
CAPBP	*Capsella bursa-pastoris* (L.) Medik.	+	−	−	−
PLAMA	*Plantago major* L.	+	−	−	−
PLAOV	*Plantago ovata* Forssk.	+	−	−	−
RBITI *	*Rubia tinctorum* L.	+	−	−	−
TRBTE	*Tribulus terrestris* L.	−	+	−	−
HEQGL	*Herniaria glabra* L.	−	−	+	−
MEDRI	*Medicago rigidula* (L.) All.	−	−	+	−

**Table 4:** Effect of treatments on the density of weeds with a significant occurrence (relative density > 3%). Those species classified as noxious weeds are indicated with an asterisk next to the EPPO code. The percentage of variation in weed density that is explained by the fixed effects of the model is indicated (*R*^2^_m_). A type III ANOVA was used to calculate the significance level of the effect (*p*-values: *** *p* < 0.001, ** *p* < 0.01, * *p* < 0.05, ns not significant), followed by a *t*-test adjusted with a Bonferroni correction for pairwise comparison. For each farm, treatment means sharing the same letter within each row are not significantly different.

	Farm A							Farm B						
	*R* ^2^_m_	*p*-Value	UC	FO	PO	PC	PL	*R* ^2^_m_	*p*-Value	UC	FO	PO	PC	PL
BROMA	0.40	***	33.67 a	1.53 b	27.17 ab	17.98 ab	1.53 b	0.32	***	17.98 a	3.44 b	9.18 ab	3.83 b	0.77 b
CHEAL *	0.09	ns	1.53 a	0.38 a	10.33 a	4.97 a	2.30 a	−	−	−	−	−	−	−
CONAR *	−	−	−	−	−	−	−	0.20	***	16.45 a	13.39 ab	12.63 ab	17.60 a	4.59 b
CYNDA *	0.16	***	4.21 b	0.77 b	21.04 a	3.83 b	6.89 ab	−	−	−	−	−	−	−
CYPRO *	0.34	**	14.54 ab	2.68 b	22.96 ab	30.61 a	0.38 b	−	−	−	−	−	−	−
DIPER *	−	−	−	−	−	−	−	0.02	ns	3.44 a	9.18 a	3.83 a	4.97 a	4.59 a
EPHCH	−	−	−	−	−	−	−	0.07	***	81.11 a	77.67 a	61.98 ab	32.52 ab	10.33 b
ERICA *	0.42	***	128.18 a	10.71 c	66.57 b	36.35 bc	3.44 c	−	−	−	−	−	−	−
FESAR	0.06	ns	30.23 a	9.57 a	26.78 a	4.21 a	1.91 a	−	−	−	−	−	−	−
GALPR	−	−	−	−	−	−	−	0.34	**	16.45 a	1.53 b	7.65 ab	1.91 b	0.00 b
MEDMI	−	−	−	−	−	−	−	0.35	*	7.27 a	6.50 a	2.68 ab	1.53 ab	0.00 b
SONAS *	0.26	**	8.80 a	0.77 b	8.80 a	3.06 ab	0.38 b	0.43	***	10.71 a	7.65 ab	3.83 bc	3.83 bc	1.15 c

**Table 5:** Correlations between living mulch cover and weed density and weed biomass, calculated for the two farms (overall) and for each farm separately. The correlation value (*r*) was estimated using a Spearman correlation test. The significance level of the correlations is also indicated (*** *p* < 0.001).

	**Overall**	
	Weed density	Weed biomass
Living mulch cover	−0.60 ***	−0.69 ***
	**Farm A**	
	Weed density	Weed biomass
Living mulch cover	−0.72 ***	−0.79 ***
	**Farm B**	
	Weed density	Weed biomass
Living mulch cover	−0.45 ***	−0.57 ***

**Table 6:** Summary table showing for each living mulch (FO, *Festuca ovina*; PO, *Pilosella officinarum*; PC, *Plantago coronopus*, and PL, *Plantago lanceolata*) the average living mulch cover, weed suppression percentage and weed biomass/weed density ratio.

	Farm A			Farm B		
	Living Mulch Cover (%)	Weed Supression (%) ^a^	Ratio (g Plants^−1^) ^b^	Living Mulch Cover (%)	Weed Supression (%) ^a^	Ratio (g Plants^−1^) ^b^
**FO**	77	93	0.31	71	61	0.61
**PO**	41	29	1.01	83	68	0.71
**PC**	33	43	1.17	54	71	0.97
**PL**	77	95	0.40	84	93	0.51

^a^ Weed biomass reduction of the living mulch with respect to the control expressed as a percentage. ^b^ Ratio of weed biomass to weed density (i.e., how much weed biomass there is per weed).

## Data Availability

Not applicable.

## Appendix A

### Biomass Estimation of Living Mulches

To estimate the biomass of living mulches, during the spring of 2022, at the time of highest vegetation vigor and after weed sampling was completed, sampling frames 0.33 × 0.33 m virtually marked on a decimal grid (i.e., 0.033 × 0.033 m) were used. Ten randomly distributed 0.033 × 0.033 m samples were taken within each frame (i.e., a 10% aliquot of the sampling frame) from each living mulch were harvested. Each sample was individually collected in a paper bag, oven-dried at 105 °C for 24 h, and subsequently weighed. Therefore, the biomass of each species was estimated, expressed in g m^−2^ (Table A1). A 1% cover measured on a 0.33 × 0.33 m frame corresponds to 1.18 g m^−2^ of FO living mulches, 0.28 g m^−2^ of PO living mulches, 0.53 g m^−2^ of PC living mulches, and 1.20 g m^−2^ of PL living mulches.

**Table A1:** Estimation of living mulch biomass from the weights (in grams) of 10 random samples (0.033 × 0.033 m) of each of the living mulches used in the study.

Samples	FO	PO	PC	PL
1	11.12	3.97	5.96	9.64
2	12.91	1.93	6.33	13.77
3	14.48	1.48	4.48	6.33
4	10.38	2.96	4.13	13.84
5	10.09	3.31	6.93	13.36
6	13.08	3.01	5.44	13.75
7	10.21	3.42	2.33	12.66
8	9.14	3.06	3.84	7.52
9	16.88	2.63	7.84	9.34
10	9.67	2.20	6.22	19.89
Mean	11.80	2.80	5.35	12.01
SE	2.48	0.75	1.65	3.93
**Biomass** (g m^−2^)	**1083.20**	**256.84**	**491.28**	**1102.85**

## Appendix B

### Sowing Cost Estimation of Two Under-Vine Living Mulches

The seed price per gram, according to the seed supplier (https://cantuesoseeds.com, accessed on 22 June 2022) is 0.040 € for *Festuca ovina* and 0.042 € for *Plantago lanceolata*. In a vineyard with a planting density of 4545 grapevines per hectare (2.0 m × 1.1 m), the sowing area would be:

1.1 m [inter-vine spacing] × 0.5 m [sowing width under-vine] = 0.55 m^2^

4544 inter-vine spaces × 0.55 m^2^ = 2499.2 m^2^ = 0.25 ha

Applying the recommended seeding rates (95,000 g ha^−1^ for *F. ovina*, 20,000 g ha^−1^ for *P. lanceolata*), the cost for one hectare of vineyard would be of 950 € for *F. ovina* (95,000 g ha^−1^ × 0.25 ha × 0.040 € g^−1^) and of 210 € for *P. lanceolata* (20,000 g ha^−1^ × 0.25 ha × 0.042 € g^−1^).
